# Supplementary material for: Multi‐dataset identification of innovative feature genes and molecular mechanisms in keratoconus
Source: J Cell Mol Med. 2024 Sep 19;28(18):e70079. doi: 10.1111/jcmm.70079 (PMC11412914; doi:10.1111/jcmm.70079)
Supplement: Supplementary file 2 — Material S2. [file JCMM-28-e70079-s002.zip › 7.GSEA analysis/4.MYRF/KEGG_AMINOACYL_TRNA_BIOSYNTHESIS.html]

Details for gene set KEGG\_AMINOACYL\_TRNA\_BIOSYNTHESIS[GSEA]

|  || Dataset | MYRF.group.cls#h\_versus\_l |
| Phenotype | group.cls#h\_versus\_l |
| Upregulated in class | h |
| GeneSet | KEGG\_AMINOACYL\_TRNA\_BIOSYNTHESIS |
| Enrichment Score (ES) | 0.2627712 |
| Normalized Enrichment Score (NES) | 0.79269 |
| Nominal p-value | 0.70866144 |
| FDR q-value | 1.0 |
| FWER p-Value | 1.0 |
Table: GSEA Results Summary

  

Fig 1: Enrichment plot: KEGG\_AMINOACYL\_TRNA\_BIOSYNTHESIS      
 Profile of the Running ES Score & Positions of GeneSet Members on the Rank Ordered List

  

| SYMBOL | TITLE | RANK IN GENE LIST | RANK METRIC SCORE | RUNNING ES | CORE ENRICHMENT || 1 | SEPSECS | na | 8099 | 0.136 | -0.0901 | Yes |
| 2 | PARS2 | na | 11719 | 0.067 | -0.1235 | Yes |
| 3 | LARS2 | na | 12120 | 0.062 | -0.0700 | Yes |
| 4 | CARS2 | na | 12424 | 0.058 | -0.0180 | Yes |
| 5 | MARS2 | na | 13887 | 0.039 | -0.0187 | Yes |
| 6 | WARS2 | na | 14376 | 0.034 | 0.0027 | Yes |
| 7 | IARS2 | na | 14818 | 0.029 | 0.0211 | Yes |
| 8 | TARS2 | na | 16176 | 0.016 | -0.0013 | Yes |
| 9 | EARS2 | na | 16578 | 0.012 | 0.0000 | Yes |
| 10 | AARS2 | na | 18092 | -0.001 | -0.0421 | Yes |
| 11 | MTFMT | na | 18803 | -0.008 | -0.0539 | Yes |
| 12 | FARSB | na | 18886 | -0.009 | -0.0468 | Yes |
| 13 | NARS2 | na | 18974 | -0.010 | -0.0387 | Yes |
| 14 | RARS2 | na | 19094 | -0.012 | -0.0300 | Yes |
| 15 | PSTK | na | 19905 | -0.021 | -0.0317 | Yes |
| 16 | HARS2 | na | 20091 | -0.023 | -0.0129 | Yes |
| 17 | FARSA | na | 21089 | -0.035 | -0.0052 | Yes |
| 18 | SARS2 | na | 21175 | -0.036 | 0.0298 | Yes |
| 19 | FARS2 | na | 23379 | -0.065 | 0.0351 | Yes |
| 20 | DARS2 | na | 24231 | -0.080 | 0.0944 | Yes |
| 21 | YARS2 | na | 24982 | -0.093 | 0.1705 | Yes |
| 22 | VARS2 | na | 25608 | -0.105 | 0.2628 | Yes |
Table: GSEA details [plain text format]

  

Fig 2: KEGG\_AMINOACYL\_TRNA\_BIOSYNTHESIS      
 Blue-Pink O' Gram in the Space of the Analyzed GeneSet

  

Fig 3: KEGG\_AMINOACYL\_TRNA\_BIOSYNTHESIS: Random ES distribution      
 Gene set null distribution of ES for **KEGG\_AMINOACYL\_TRNA\_BIOSYNTHESIS**
